# Supplementing a *Saccharomyces cerevisiae* fermentation product modulates innate immune function and ameliorates bovine respiratory syncytial virus infection in neonatal calves

**DOI:** 10.1093/jas/skaa252

**Published:** 2020-08-11

**Authors:** Asmaa H A Mahmoud, Jamison R Slate, Suyeon Hong, Ilkyu Yoon, Jodi L McGill

**Affiliations:** 1 Department of Veterinary Microbiology and Preventive Medicine, Iowa State University, Ames, IA; 2 Agricultural Research Center, Animal Health Research Institute, Giza, Egypt; 3 Diamond V Mills Inc., Cedar Rapids, IA

**Keywords:** bovine respiratory disease, bovine respiratory syncytial virus, innate immunity, *Saccharomyces cerevisiae* fermentation products

## Abstract

The objectives of this study were to determine the effects of oral supplementation with *Saccharomyces cerevisiae* fermentation products (**SCFP**; SmartCare and NutriTek; Diamond V, Cedar Rapids, IA) on immune function and bovine respiratory syncytial virus (**BRSV**) infection in preweaned dairy calves. Twenty-four Holstein × Angus, 1- to 2-d-old calves (38.46 ± 0.91 kg initial body weight [**BW**]) were assigned two treatment groups: control or SCFP treated, milk replacer with 1 g/d SCFP (SmartCare) and calf starter top-dressed with 5 g/d SCFP (NutriTek). The study consisted of one 31-d period. On days 19 to 21 of the supplementation period, calves were challenged via aerosol inoculation with BRSV strain 375. Calves were monitored twice daily for clinical signs, including rectal temperature, cough, nasal and ocular discharge, respiration effort, and lung auscultation. Calves were euthanized on day 10 postinfection (days 29 to 31 of the supplementation period) to evaluate gross lung pathology and pathogen load. Supplementation with SCFP did not affect BW (*P* = 0.762) or average daily gain (*P* = 0.750), percentages of circulating white blood cells (*P* < 0.05), phagocytic (*P* = 0.427 for neutrophils and *P =* 0.460 for monocytes) or respiratory burst (*P* = 0.119 for neutrophils and *P =* 0.414 for monocytes) activity by circulating leukocytes either before or following BRSV infection, or serum cortisol concentrations (*P* = 0.321) after BRSV infection. Calves receiving SCFP had reduced clinical disease scores compared with control calves (*P =* 0.030), reduced airway neutrophil recruitment (*P* < 0.002), reduced lung pathology (*P* = 0.031), and a reduced incidence of secondary bacterial infection. Calves receiving SCFP shed reduced virus compared with control calves (*P* = 0.049) and tended toward lower viral loads in the lungs (*P* = 0.051). Immune cells from the peripheral blood of SCFP-treated calves produced increased (*P* < 0.05) quantities of interleukin (**IL**)-6 and tumor necrosis factor-alpha in response to toll-like receptor stimulation, while cells from the bronchoalveolar lavage (**BAL**) of SCFP-treated calves secreted less (*P* < 0.05) proinflammatory cytokines in response to the same stimuli. Treatment with SCFP had no effect on virus-specific T cell responses in the blood but resulted in reduced (*P* = 0.045) virus-specific IL-17 secretion by T cells in the BAL. Supplementing with SCFP modulates both systemic and mucosal immune responses and may improve the outcome of an acute respiratory viral infection in preweaned dairy calves.

## Introduction

Respiratory disease is the leading cause of morbidity and mortality among feedlot cattle, the second leading cause of mortality in preweaned dairy calves, and the most common cause of weaned dairy heifer mortality (Sacco et al., 2012a, 2014). Economic costs to the cattle industry due to respiratory disease have been estimated as high as US $3 billion annually due to death losses, reduced performance, and costs of vaccinations and treatment (Watts and Sweeney, 2010). Despite the widespread use of prevention and control measures, there has been little progress in reducing the prevalence or costs associated with bovine respiratory disease (**BRD**) during the last two decades (USDA, 2010, 2012).

BRD is typically initiated by a primary viral infection that subsequently predisposes the animal to exacerbated, secondary bacterial pneumonia (Taylor et al., 2010). Bovine respiratory syncytial virus (**BRSV**) is amongst the most common viral pathogens that contributes to BRD. Infection with BRSV is not only most severe in calves less than 6 mo of age but can also affect older cattle. The worldwide frequency of exposure to BRSV infection in beef and dairy herds reportedly exceeds 50% (Gershwin, 2007) and may be as high as 70% to 100% (Ohlson et al., 2013). Following the outbreaks of BRD, rates of seroconversion to BRSV can reach up to 45% (Brodersen, 2010). In the instance of enzootic calf pneumonia, BRSV is a documented cause in up to 60% of the cases (Meyer et al., 2008).

Given concerns related to the development of antimicrobial resistance and the increased demand by consumers to reduce antibiotic residues in animal products, there is interest in identifying alternative strategies, which can be used to prevent or reduce the impact of BRD. *Saccharomyces cerevisiae* fermentation products (**SCFP**) are produced through a proprietary anaerobic fermentation process and are comprised of a complex blend of bioactives, including amino acids, organic acids, polyphenols, and lipid and B vitamins. Supplementation of SCFP has been shown to have positive impacts on performance, health, and immunity in humans and multiple animal species, including swine, poultry, and cattle (Moyad et al., 2009; Shen et al., 2011; Brewer et al., 2014; Alugongo et al., 2017). In preweaned calves, SCFP added to the milk and starter grain have beneficial effects on rumen development and the gut microbiota and improve the outcome of *Salmonella enterica* challenge and *Cryptosporidium parvum* infection (Brewer et al., 2014; Vázquez Flores et al., 2016; Vélez et al., 2019). Anecdotal reports from the field have suggested that SCFP supplementation may also improve the outcome of BRD in young calves, in dairy heifers when transitioning to group pens and freestalls, and in feedlot animals. However, to date, there have been no studies investigating the impact of SCFP supplementation on respiratory health or susceptibility to respiratory infection in the bovine. Therefore, the objectives of this study were to determine the impact of SCFP supplementation on local and systemic immune function and to determine the effect of SCFP treatment on susceptibility to BRSV infection in preweaned dairy calves. We hypothesized that supplementing SCFP during the neonatal period would modulate innate and adaptive immune function and reduce the severity of BRSV challenge.

## Materials and Methods

The experimental procedures were approved by the Iowa State University Institutional Animal Care and Use Committee (protocol 19-202) and the Institutional Biosafety Committee (protocol 19-119).

### Animal care and feeding

The study was conducted as a randomized complete block design consisting of one 31-d period, with 24 Holstein × Angus mixed sex (10 bulls, 14 females, 38.46 ± 0.91 kg initial body weight [**BW**]) calves. Calves were enrolled in the study at 1 to 2 d of age. The animals were purchased from a commercial dairy located in western Iowa and transported approximately 2 h to the Livestock Infectious Disease Isolation Facility at Iowa State University. Enrollment criteria were based upon a satisfactory health assessment upon arrival and adequate passive transfer. A serum sample was collected upon arrival and immediately tested for passive transfer status using the Immunoglobulin G (IgG) Check calf side passive-transfer kit (PortaCheck Inc., Moorestown, NJ). The animals were blocked by initial BW and then randomly divided into two treatment groups: 1) control: base milk replacer and calf starter and 2) SCFP supplemented. The control group consisted of eight females and four males. The SCFP fed group consisted of six females and six males. The SCFP products, SmartCare and NutriTek, were supplied by Diamond V. SmartCare was fed at a rate of 1 g/d mixed into the milk replacer (0.5 g/feeding), and NutriTek was fed at a rate of 5 g/d, top-dressed onto the calf starter. The calves remained on the dietary treatments for the duration of the study (31 d).

The calves were housed indoors in the environmentally controlled, BSL-2Ag facility for the duration of the study. The animals were housed individually on raised platforms. Groups of four to eight calves were housed per room in a total of five rooms. Four rooms contained four calves each and were divided by treatment (control or SCFP treated). One large room housed eight calves, with four calves on the control diet and four calves on SCFP treatment. All calves were provided ad libitum access to freshwater, using automatic watering systems, and ad libitum access to calf starter. The starter was offered in individual plastic pails. Starter was weighed each morning at 0800 hours, and daily consumption was recorded. Leftover starter was discarded, the pail cleaned as needed, and fresh calf starter was replaced. Animals were initially offered a 0.23 kg starter per day, which was increased as needed to maintain ~15% refusal rate. The base calf starter was pelleted and free of yeast products and ionophores. The ingredient and nutrient composition are presented in Table 1. For animals receiving SCFP supplementation, 5 g/d NutriTek was top-dressed onto the starter each morning.

**Table 1.** Milk replacer and calf starter formulations for calves supplemented with or without SCFP products for 31 d^1^

Ingredient^2^, % DM	Milk replacer^3^	Starter^4^
Nutrient composition, DM basis		
Dry matter, %	97	94
Crude protein, %	22	19
Fat, %	20	2.8
ADF, %	0.15	9
Calcium, %	0.78	1.7
Phosphorus, %	0.632	0.58
Vitamin A, added IU/kg	44,000	36,300
Vitamin D, added IU/kg	16,500	11,000
Vitamin E, added IU/kg	294	330

^1^Calves fed SCFP received 1 g/d SmartCare in milk and 5 g/d NutriTek top-dressed on the starter for the duration of the study.

^2^ADF, acid detergent fiber; DM, dry matter.

^3^Milk Products, Chilton, WI.

^4^Effingham Equity, Effingham, IL.

Animals were given milk replacer twice per day, at approximately 0800 and 1800 hours, and consumption was recorded at each feeding. The animals were fed from nipple bottles for the duration of the study. The milk replacer was custom formulated to contain no yeast or ionophores. The ingredient and nutrient composition are presented in Table 1. Calves were fed at a rate of 1 kg solids/d and 15.5% solids. For animals receiving SCFP supplementation, 1 g/d (0.5 g/feeding) was added to individual bottles and shaken to ensure thorough distribution.

### Blood collection, peripheral blood mononuclear cell preparation, and cryopreservation

Peripheral blood (up to 30 mL) was collected at approximately 0600 hours (before feeding) on days 1, 7, 14, and 19 during the feeding period and on days 3, 7, and 10 after BRSV infection. Blood was collected into 2× acid citrate dextrose via the jugular vein using a syringe. A fraction of the whole blood was immediately processed for flow cytometry to determine the frequencies of circulating immune populations. The second fraction of whole blood was used to evaluate leukocyte respiratory burst and phagocytic activities. The remaining whole blood was used to isolate peripheral blood mononuclear cells (**PBMCs**). Cells were isolated from buffy coats by density centrifugation as previously described (McGill et al., 2016). Contaminating red blood cells were removed using hypotonic lysis. Cells were washed twice, counted, and resuspended in complete Roswell Park Memorial Institute (**cRPMI**) composed of RPMI-1640 (Gibco, Carlsbad, CA) supplemented with 2 mM l-glutamine, 25 mM hydroxyethyl piperazineethanesulfonic acid buffer, 1% antibiotic-antimycotic solution, 1% nonessential amino acids, 2% essential amino acids, 1% sodium pyruvate, 50 μM 2-mercaptoethanol (all from Sigma, St. Louis, MO), and 10% (v/v) fetal bovine sera (**FBS**). Blood cells were cryopreserved as follows: briefly, PBMCs were resuspended at 2 × 10^7^ cells/mL in 1 mL of precooled FBS containing 10% dimethyl sulfoxide (**DMSO**), and rapidly brought to −80 °C in polystyrene containers, which ensured a slow drop in temperature. After 24 h, the cryovials were transferred to a liquid nitrogen tank where they remained until analysis.

### Bronchoalveolar lavage

Antemortem bronchoalveolar lavage (**BAL**) fluid samples were collected on day 14 of the feeding period, using a protocol we have previously described (Guerra-Maupome et al., 2019). A modified stallion catheter was blindly passed through the nose and advanced through the trachea until lodging in the bronchus. A total of 180 mL of sterile saline was divided into three aliquots. An aliquot was introduced to the lower respiratory tract, followed by immediate suction to obtain lower airway washes. The procedure was repeated twice more. All three aliquots were pooled at the end of the procedure. BAL samples were kept on ice, filtered over sterile gauze, and centrifuged at 200 × *g* for 10 min. Contaminating red blood cells were removed using hypotonic lysis. Cells were washed, counted, and resuspended in cRPMI for subsequent assays or cryopreservation.

### Leukocyte respiratory burst and phagocytosis assays

Cleavage of dihydrorhodamine(**DHR**)-123 was used to determine the leukocyte respiratory burst activity. Fresh, whole blood samples were loaded with 0.1 µM DHR by incubating for 20 min in a 37 °C water bath. Cells were then stimulated or not with 0.2 µM Phorbol 12-myristate 13-acetate (20 min in a 37°C water-bath). The reaction was stopped by placing cells on ice. Samples were then surface stained for flow cytometry to identify monocytes (CD14^+^) and granulocytes (CH138^+^).

Leukocyte phagocytic activity was determined in whole blood samples using a commercial Phagocytosis Assay Kit (IgG FITC, Caymen Chemical). Fresh whole blood samples were incubated with a 1:200 dilution of latex beads coated with fluorescently labeled rabbit IgG. Samples were incubated for 2 h in a 37 °C water bath. Samples were then surface stained for flow cytometry to identify monocytes (CD14^+^) and granulocytes (CH138^+^).

### Performance data

Calf starter and milk replacer intake were recorded daily. Calves were weighed upon arrival, once per week during the feeding period, once prior to BRSV infection, and once immediately prior to necropsy.

### BRSV infection

Calves were infected with BRSV on days 19 to 21 of the feeding period. For logistical purposes, the viral challenges were staggered by 24 h, with 7 to 8 calves/d receiving the virus, balanced by treatment. Animals were challenged via aerosol inoculation with ~10^4^ Tissue Culture Infectious Dose (TCID)_50_ BRSV strain 375 as we have previously described (McGill et al., 2016, 2018, 2019a). Briefly, the viral inoculum was suspended in 5 mL and delivered via forced-air nebulizer to a mask covering the nose and mouth of the calf. The viral inoculum was prepared by re-isolating virus from the lungs of an infected calf. The virus stock was passaged twice on primary bovine turbinate cells, processed through two freeze-thaw cycles, and clarified by centrifugation. The stock was bottled and stored at −80 °C until infection. The virus stock was confirmed free of BVDV, and the TCID_50_ was determined by titration on bovine turbinate cells.

### Clinical illness scoring

Calves were monitored twice daily for clinical illness by a single trained observer who was blinded to treatments. Calves were scored using an adaptation of the University of Wisconsin Calf Health Respiratory Scoring Chart, originally established by Dr. Sheila McGuirk (https://www.vetmed.wisc.edu/fapm/svm-dairy-apps/calf-health-scorer-chs/). The scoring chart assigns numbers (0 to 3) based upon rectal temperature (0 = 100 to 100.9, 1 = 101 to 101.9, 2 = 102 to 102.9, and 3 = >103) and severity of clinical signs, including cough (0 = none, 1 = induced single cough, 2 = induce repeated cough or spontaneous cough, 3 = repeated, spontaneous coughing), nasal discharge or ocular discharge (0 = normal, 1 = small amount of mild discharge, 2 = moderate, bilateral discharge, 3 = copious, bilateral discharge), and ear position (0 = normal, 1 = ear flicking, 2 = slight unilateral droop, 3 = severe head tilt or bilateral ear droop). Our scoring chart includes an additional category for respiratory effort (0 = no effort to 3 = significant effort) and for lung auscultation (0 = clear lungs; 1 = wheezing or other harsh lung sounds).

### Virus isolation

Nasal swabs were collected from each calf on days 0, 1, 3, 7, and 10 postinfection and placed in virus isolation media (serum-free minimal essential medium [**MEM**] with antibiotics). Swabs were immediately frozen at −80 °C and stored until analysis. Virus isolations were performed as previously described (Sacco et al., 2012b). Supernatants from nasal swabs or lung homogenates were inoculated onto confluent monolayers of Madin–Darby Bovine Kidney cells and incubated for 90 min at 37 °C with 5% CO_2_. After incubation, the inoculum was aspirated and fresh MEM containing 10% FBS was added. Cultures were incubated at 37 °C, 5% CO_2_ for 7 d. The cytopathic effect was observed and recorded daily.

### Necropsy and pathological evaluation

Calves were euthanized on day 10 postinfection by barbiturate overdose. Pathological evaluation was performed similar to previous descriptions (Viuff et al., 2002; Sacco et al., 2012b), and the extent of pneumonic consolidation was evaluated using the scoring system that we have published (McGill et al., 2018): 0 = free of lesions; 1 = 1% to 5% affected; 2 = 6% to 15% affected; 3 = 16% to 30% affected; 4 = 31% to 50% affected; and 5 = >50% affected. Samples of affected and unaffected lung tissue were collected and stored for virus isolation and qPCR analysis. Samples of the affected lung were also submitted to the Iowa State University Veterinary Diagnostic Laboratory for testing with the Bovine Respiratory Viral and Bacterial polymerase chain reaction (PCR) panel (tests for bovine viral diarrhea virus, BRSV, bovine parainfluenza virus, bovine coronavirus, bovine herpesvirus-I, *Mannheimia haemolytica, Pastuerella multocida*, and *Mycoplasma bovis*) and culturing.

Postmortem BAL fluid was collected at necropsy by removing the lungs and trachea and introducing 500 mL of sterile, ice-cold saline through the trachea. The lungs were massaged, and then fluid was poured back out of the trachea into sterile collection bottles. Cells from the BAL were filtered, and red blood cell (RBC) lysis was performed as described above. Fresh BAL samples were submitted to the Iowa State University Veterinary Diagnostic Laboratory for the preparation of cytospins and differential staining (Modified Wrights Stain). Cytology was performed by a blinded individual, and a minimum of 500 cells were counted per sample. Duplicate samples were prepared for each animal.

### Real-time PCR

qPCR for the BRSV NS2 gene was performed on nasal swabs and lung tissue to determine viral load. Ribonucleic acid isolation was performed as described (McGill et al., 2018). NS2 qPCR was performed using the Taqman RNA-to-CT 1-step kit (Applied Biosystems) as in McGill et al. (2018). Viral NS2 copy numbers were calculated using standard curves. For lung tissue, NS2 copy numbers were normalized to the S9 housekeeping gene to correct for differences in the input material. qPCR was run on a ThermoFisher Scientific QuantStudio 3 Real-Time PCR machine.

### Thawing and in vitro cell stimulation

For thawing, PBMCs or alveolar macrophages were removed from the liquid nitrogen and thawed in a 37 °C water bath for 2 min. Once thawed, the cells were rapidly transferred to 15-mL polystyrene tubes containing 8 mL of warm cRPMI to remove DMSO. Finally, the cells were washed twice with complete medium and counted. Cell viability was assessed in each sample using the Trypan Blue method and found to be >85%.

For toll like receptor-agonist (innate) stimulation experiments, PBMCs or BAL cells (1 × 10^6^ cells/mL, 1 mL/well) were added to 24-well, tissue-culture-treated plates and rested overnight at 37 °C, 5% CO_2_. Cell cultures were then washed once with warm cRPMI, and the cells were stimulated with cRPMI (negative control), 1 µg/mL lipopolysaccharide (**LPS**), 10 µg/mL Pam3CSK4, or a mixture of 50 μg/mL Poly(I:C) with 10 μg/mL imiquimod (all purchased from InvivoGen). Cells were incubated for 72 h at 37 °C, 5% CO_2_. Cell culture supernatants were collected and stored at −80 °C.

To evaluate the virus-specific adaptive immune response, cells were thawed and rested as above, then used for either cell proliferation assays or intracellular staining. For proliferation assays, PBMCs were labeled using CellTrace Violet (Invitrogen, Carlsbad, CA) following the manufacturer’s instructions. Briefly, PBMCs were resuspended at 1 × 10^7^cells/mL in phosphate buffered saline (PBS) containing 5 μM/mL of the CellTrace dye. After gently mixing, PBMCs were incubated for 20 min at 37 °C in a water bath. Labeling was quenched by using an equal volume of FBS, and cells were washed three times with RPMI medium. Subsequently, cells (5 × 10^5^/well) were plated in round-bottom 96-well plate in duplicates, cultured for 6 d at 37 °C, 5% CO_2_, with 0.01 multiplicity of infection (MOI) BRSV strain 375. Concanavalin A (5 µg/mL) and cRPMI medium were added to positive and negative control wells, respectively. After 6 d, cell culture supernatants were collected and stored at −80 °C, and cells were surface stained and analyzed for proliferation and surface marker expression by flow cytometry.

For intracellular cytokine staining, 1 × 10^6^ PBMCs or BAL cells were incubated in cRPMI containing 0.01 MOI BRSV strain 375 for 16 h at 37 °C, 5% CO_2_ with brefeldin A (GolgiPlug; BD Pharmingen, 10 μg/mL) added during the last 4 h of culture. After staining cell-surface markers (see the Flow cytometry section), cells were fixed and permeabilized for 20 min using BD CytoFix/CytoPerm solution (BD Biosciences) and incubated with anti-bovine interferon-gamma (**IFNγ**)-PE (Table 2) for 30 min. Non-stimulated samples served as negative controls. Concanavalin A-stimulated samples served as positive controls.

**Table 2.** Flow cytometry reagents

Reagent or antibody clone	Specificity, Source	Secondary antibody, Source
ILA11	Bovine CD4, Kingfisher Biotech, Inc.	Alexafluor-488, Life Technologies
GB21A	Bovine TCR1 delta chain, Kingfisher Biotech, Inc.	PE-Cy7, SouthernBiotech
CACT80C	Bovine CD8, Kingfisher Biotech, Inc.	Allophycocyanin, Life Technologies
CH138	Bovine granulocytes, Kingfisher Biotech, Inc.	Allophycocyanin, Life Technologies
CAM36A	Bovine CD14, Kingfisher Biotech, Inc.	PE, Life Technologies
AKS1	Bovine CD335-PE, BioRad	Not applicable
CC302	Bovine IFN-γ-PE, BioRad	Not applicable
CellTrace Violet	Not applicable, Life Technologies	Not applicable

To measure virus-specific cytokine secretion by BAL cells, samples were plated (1 × 10^6^ cells/mL, 1 mL/well, 24-well plate) and rested overnight. Cells were then washed once with warm cRPMI, and then stimulated for 72 h with cRPMI (negative control), 0.01 MOI BRSV strain 375, or 5 μg/mL Concanavalin A as a positive control. Cell culture supernatants were then collected and stored at −80 °C.

### Flow cytometry

Fresh whole blood was processed for flow cytometry by lysing RBC by hypotonic lysis, washing, and then surface staining. For other cell types, following the appropriate culture duration, cells were centrifuged, cell culture supernatants were removed and stored, and cells were then stained with Live/Dead Aqua (Thermofisher) and primary and secondary monoclonal antibodies listed in Table 2. All incubation steps for staining were performed in flourescence activated cell sorting (FACS) buffer (PBS with 10% FBS and 0.02% NaN_3_) and incubated for 20 min at 4 °C. Cells were washed and fixed with BD FACS lysis buffer (BD Biosciences, Mountain View, CA) for 10 min at room temperature, and then washed and resuspended in FACS buffer until analysis. Samples were acquired using a BD FACS Canto flow cytometer (BD Biosciences). Data were analyzed using Flowjo software (Tree Star Inc., San Carlos, CA). For antigen recall assays, lymphocytes were identified in PBMCs and BAL samples as shown in Supplementary Figures S1–S3 and were further subdivided by the expression of CD4 and CD8.

### Enzyme-linked immunosorbent assay

Cell culture supernatants were stored at −80 °C until enzyme-linked immunosorbent assay (ELISA) analysis. The concentration of cytokines in cell culture supernatants was determined using bovine commercial ELISA kits for IFNγ (intra-assay coefficient of variation [CV] 3.6% and inter-assay CV 6.1%), interleukin (**IL**)-17A (intra-assay CV 6.7% and inter-assay CV 7.6%), IL-6 (intra-assay CV 4.1% and inter-assay CV 5.0%), and TNFα (intra-assay CV 4.3% and inter-assay CV 17.0%) from Kingfisher Biotech, Inc. Minneapolis, MN. Bovine IL-1β was measured using a commercial ELISA kit (intra-assay CV 4.6% and inter-assay CV 7.7% as per manufacturer) from ThermoFisher Scientific according to the manufacturer’s instructions. Each sample was assayed in duplicate.

Serum (up to 8 mL) was collected on days 0, 3, 7, and 10 after infection using SST Serum Separator Tubes (BD Vacutainer). Serum cortisol was determined using a commercial Cortisol enzyme immunoassay kit per manufacturer’s instructions (Arbor Assays, Ann Arbor, MI; intra-assay CV 8.7% ± 5.1% and inter-assay CV 8.1% ± 2.4% as per manufacturer). Each sample was assayed in duplicate.

### Statistics

Data were graphed and statistical analyses were performed using GraphPad Prism v8.3.1 (Graphpad Software, Inc.). Experiments were analyzed as a randomized complete block design. Individual calf was considered the experimental unit. The results were tested for normal distribution prior to data analysis using the D′Agostino-Pearson normality test. For data in which assessments were performed on multiple days, results were analyzed by a linear mixed-effects model, fit using restricted maximum likelihood with the Geisser–Greenhouse correction, followed by Sidak’s test for multiple comparisons. The model used the fixed effects of time, treatment (control vs. SCFP diet), and treatment × time interaction. Calf was considered a random effect. Single measurement data were analyzed by one-way analysis of variance, unpaired *t*-test, or Mann–Whitney test, as indicated in the respective figure legends. Data are reported as least squares means ± SEM. Differences of *P* ≤ 0.05 were considered significant, and tendencies were declared at 0.05 < *P* ≤ 0.10.

## Results

### Calf losses

Two calves in the control group died during the feeding period. Both calves developed watery scours and dehydration, further complicated by the symptoms of respiratory disease. Both calves were treated with a two-dose course of ceftiofur and received supportive care for dehydration (electrolytes, intravenous fluids, and bismuth subsalicylate). The animals failed to recover and were euthanized for humane reasons. The first calf was euthanized on day 12 during the feeding period. A necropsy was performed and revealed that enteric infection caused by *C. parvum* and *Clostridium perfringens*, and a moderate bronchopneumonia caused by *M. haemolytica.* The second animal was euthanized on day 20 during the feeding period. A necropsy revealed *C. parvum* infection, with a moderate bronchopneumonia caused by *H. somni* and *M. bovis.* The data from these two calves are included in the intake and performance analyses, and immunology analyses, until ceftiofur treatment was initiated, after which the animals are excluded.

### Performance

**Figure 1.**
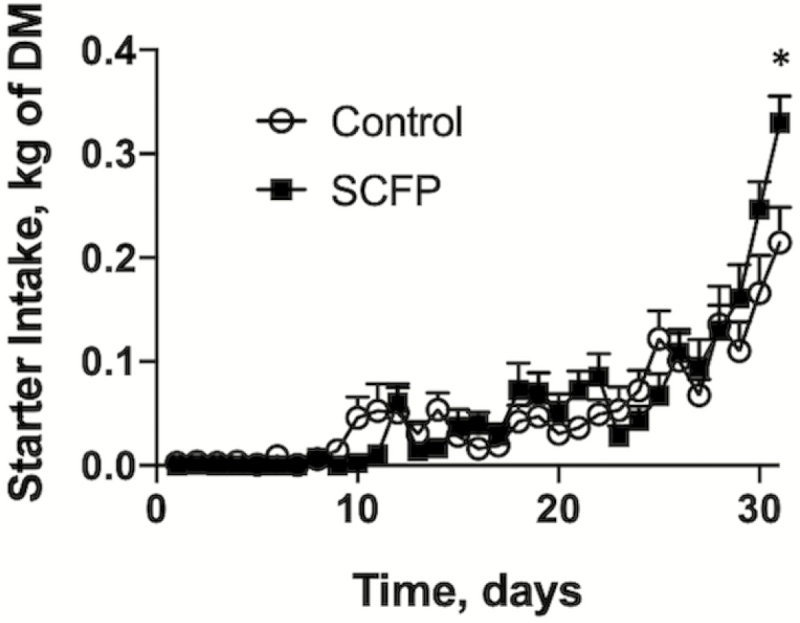
The effects of SCFP supplementation on calf starter intake on days 1 to 31. Calf starter intake was monitored daily in control (*n* = 12) and SCFP-treated calves (*n* = 12) for the duration of the study. One control calf was removed from the study on day 12. A second control animal was removed from the study on day 20. The remaining calves were challenged via aerosol inoculation with BRSV strain 375 on days 19 to 21. One control calf was euthanized for humane reasons on day 8 postinfection (day 27). Data are presented as means ± SEM. We noted a treatment × time interaction (*P* < 0.0001).

We observed no differences in the starting or final BW between treatment groups (Table 3). Overall starter intake (from days 1 to 31) was similar between treatment groups (0.051 and 0.052 kg/d for control and SCFP, respectively). We did, however, observe a treatment × time interaction in calf starter intake (*P* < 0.0001; Table 3; Figure 1). Calves supplemented with SCFP increased starter intake as they grew older. The average daily gain was not different between treatment groups (*P* = 0.750). No effects (*P =* 0.805) on milk replacer intake were observed between controls and SCFP-treated animals.

**Table 3.** Effects of treatments on performance measurements in calves supplemented with or without SCFP products for 31 d

Parameter	Control mean	SCFP^1^ mean	SEM	Treatment effect *P-*value	Time effect *P-*value	Treatment × Time interaction *P-*value
Initial BW^2^, kg	39.83	39.10	1.36	0.635		
Final BW, kg	54.62	55.25	1.63	0.762		
Starter intake, kg/d	0.051	0.052	0.013	0.446	<0.0001	<0.0001
Average daily gain^3^, kg/d	0.485	0.503	0.051	0.750	<0.0001	0.123

^1^Calves fed SCFP received 1 g/d SmartCare in milk and 5 g/d NutriTek top-dressed on the starter for the duration of the study.

^2^BW were collected upon arrival, once weekly during the feeding period, once prior to BRSV infection, and once on the day of necropsy.

^3^Average daily gain was calculated based on intake from 29 to 31 d, dependent upon when the animal was euthanized.

### BRSV infection

**Figure 2.**
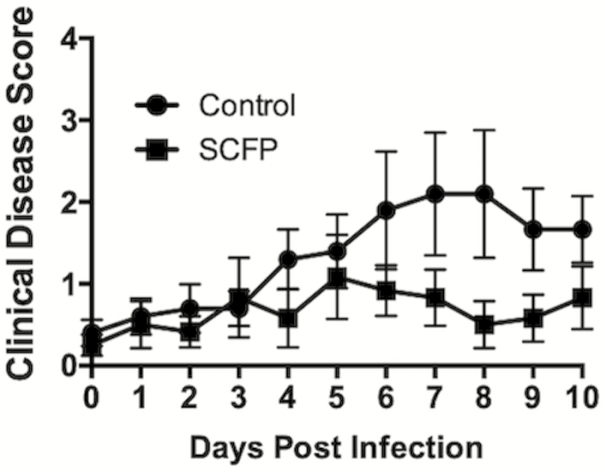
SCFP treatment reduces BRSV-associated clinical disease. Calves in the control group (*n* = 10) and SCFP-treated group (*n* = 12) were challenged via aerosol inoculation with BRSV strain 375 on days 19 to 21 of the study. (A) Clinical signs were monitored twice daily by a single trained observer and scores were assigned to each animal once daily using the scoring system described in Materials and Methods. Scoring parameters include body temperature, respiration rate, ocular and/or nasal discharge, cough, and lung auscultation. One animal in the control group was euthanized on day 8 postinfection for humane reasons. Clinical disease scores were reduced in the SCFP-treated group (treatment effect, *P* < 0.03). Data are presented as means ± SEM.

**Figure 3.**
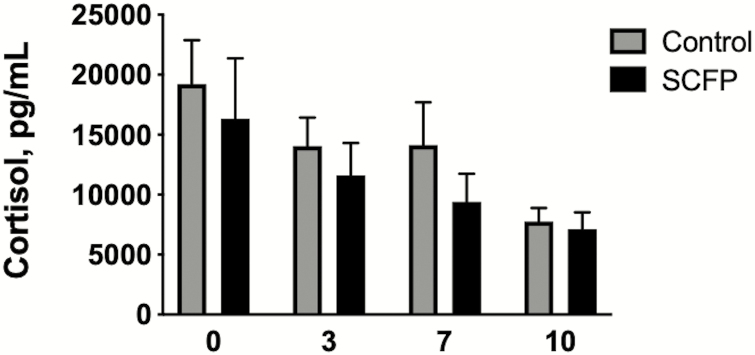
Serum cortisol levels following BRSV infection. Calves in the control group (*n* = 10) and SCFP-treated group (*n* = 12) were challenged via aerosol inoculation with BRSV strain 375 on days 19 to 21 of the study. Serum samples were collected on days 0, 3, 7, and 10 after infection and analyzed for cortisol concentrations using a commercial ELISA kit. One calf in the control group was euthanized on day 8 postinfection and is not included in the results for day 10 after infection. Data are presented as means ± SEM. Treatment effect, *P =* 0.321; Time effect, *P =* 0.014; Treatment × Time interaction, *P =* 0.913.

The BRSV infection caused mostly mild respiratory disease in the control calves, with elevated body temperatures, occasional coughing, and increased respiratory effort. Clinical disease symptoms were sustained for approximately 3 to 4 d, peaking in the control calves on days 7 to 8 after infection (Figure 2). One control calf developed more severe disease and was euthanized for humane reasons on day 8 postinfection. The severity of BRSV infection was reduced (treatment effect, *P* = 0.03) in the SCFP-treated calves, resulting in only mild and transient disease signs (Figure 2). In these animals, the clinical disease score peaked around days 5 to 6 after infection and then returned to baseline by day 8 postinfection. Serum cortisol concentrations were monitored on days 0, 3, 7, and 10 postinfection (Figure 3). The cortisol concentration was highest on the day of challenge, then decreased over time. Serum cortisol concentrations did not vary between controls and SCFP-treated calves (*P =* 0.321).

**Figure 4.**
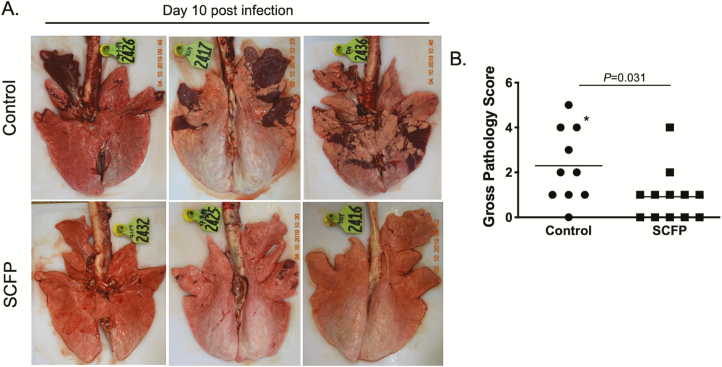
SCFP treatment reduces BRSV-associated lung pathology. Calves in the control group (*n* = 10) and SCFP-treated group (*n* = 12) were challenged via aerosol inoculation with BRSV strain 375 on days 19 to 21 of the study. The animals were humanely euthanized and necropsied on day 10 postinfection. One animal in the control group was euthanized on day 8 postinfection for humane reasons. The extent of gross pneumonic consolidation was evaluated based upon the percent of lung affected (0 = free of lesions; 1 = 1% to 5% affected; 2 = 6% 15% affected; 3 = 16% to 30% affected; 4 = 31% to 50% affected; 5 = >50% affected). (A) depicts representative images of gross lesion from three individual animals in the control group (top) or SCFP-treated group (bottom). (B) depicts aggregate gross pathology results from all animals. *Indicates the animal that was euthanized on day 8 postinfection. Data are presented as means ± SEM. *P =* 0.031 as determined by Mann–Whitney test.

The remaining calves were euthanized on day 10 after infection and a necropsy was performed. The control calves developed lung lesions that were consistent with our previous studies (Sacco et al., 2012a, 2014; McGill et al., 2018, 2019a, 2019b), with bilateral, multifocal to coalescing areas of pneumonic consolidation. Consistent with the observed reduction in clinical disease scores, SCFP-treated calves had reduced gross lung pathology (*P =* 0.031), with only a few foci of consolidation present, mostly in the cranial regions of the lung. Representative images of lungs from three control animals (top) and three SCFP-treated animals (bottom) are depicted in Figure 4A. Gross pathology scores were assigned to each animal based upon the area of consolidation (McGill et al., 2018, 2019a). As seen in Figure 4B, calves receiving SCFP treatment had reduced lung pathology scores compared with control calves (*P* = 0.031, Figure 4B).

**Figure 5.**
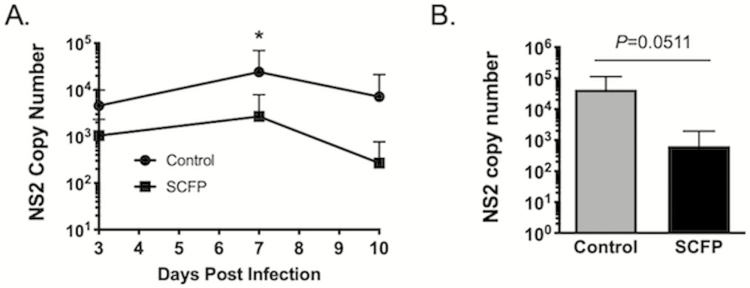
Reduced virus shedding and reduced viral burden in SCFP-treated calves compared with controls. (A) Nasal swabs were collected from all animals (*n* = 10 controls, *n* = 12 SCFP-treated calves) on days 0, 1, 3, 7, and 10 and snap-frozen at −80 °C until analysis. Swabs were analyzed by qPCR for the BRSV NS2 gene. NS2 mRNA was undetectable on day 0 of the infection. Results from days 3, 7, and 10 are depicted in the graph. Only 6/10 control calves and 3/12 SCFP-treated calves were positive for viral RNA on day 10 postinfection. Data are presented as means ± SEM. *P = 0.049. (B) The animals were humanely euthanized on day 10 postinfection. Sections of the healthy and pneumonic lung were collected from 2 to 3 representative lesion sites of the lungs and preserved in RNALater. The RNA was extracted using Trizol reagent and samples from multiple sites were then pooled and analyzed by qPCR for the BRSV NS2 gene. NS2 copy number was normalized to the housekeeping gene, S9, to correct for differences in the input material. Statistical significance was interrogated by student’s *t-*test.

Virus was isolated with similar frequency from the nasal swabs of control and SCFP-treated calves on days 3 and 7 after infection (Table 4), although the length of shedding was reduced with SCFP-treated calves, as only one SCFP-treated animal was still shedding BRSV on day 10 compared with approximately half of the control calves (Table 4). Virus was quantified in the nasal swabs and lungs using qPCR for the BRSV NS2 gene. As seen in Figure 5A, SCFP-treated calves shed the overall reduced quantities of virus on day 7 (*P* = 0.049). Because BRSV infection can predispose calves to the development of bacterial pneumonia, it is not uncommon for calves with respiratory disease to present with multiple pathogens of the BRD complex. Therefore, in addition to testing for BRSV infection, lung tissue samples were also evaluated by multiplex PCR for other common BRD-associated pathogens. Four calves in the control group tested positive by PCR for *P. multocida* and BRSV in lung tissue, although *P. multocida* could be cultured out of only one animal. One calf tested positive by PCR for *P. multocida, M. bovis*, and BRSV. In the SCFP-treated group, one calf tested positive for both *P. multocida* and BRSV, and one calf was positive for *P. multocida*, *M. bovis*, and BRSV. Neither animal was positive for *P. multocida* by culture. Besides BRSV, none of the animals were PCR positive for any other BRD-associated viral pathogens (coronavirus, bovine viral diarrhea virus, and bovine herpesvirus 1). Thus, SCFP treatment also reduced the incidence of secondary bacterial invaders following the BRSV challenge.

**Table 4.** Virus isolation results from the nasal swabs and lungs after BRSV challenge of calves supplemented with or without SCFP products for 31 d

	Virus isolation results					
	Nasal swabs					Lung tissue^3^
Treatment	Day 0	Day 1	Day 3	Day	Day 10	Day 8 or 10
Control	0/10 (0%)	0/10 (0%)	4/10 (40%)	9/10 (90%)	5/9^2^ (55.5%)	4/10 (40%)
SCFP^1^	0/12 (0%)	0/12 (0%)	5/12 (41.6%)	6/12 (50%)	1/12 (8.3%)	2/12 (17%)

^1^Calves fed SCFP received 1 g/d SmartCare in milk and 5 g/d NutriTek top-dressed on the starter for 31 d.

^2^One calf in the control group was euthanized on day 8 postinfection for humane reasons. Nasal swabs were not available for collection on day 10 postinfection.

^3^These data include the results from the calf in the control group that was euthanized on day 8 postinfection for humane reasons. The lung tissue from this animal was positive for BRSV.

**Figure 6.**
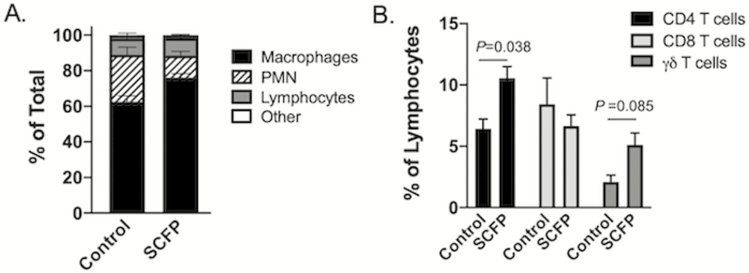
Altered leukocyte profiles in the BAL of SCFP-treated calves compared with controls. BAL samples were collected from all calves (SCFP treated, *n* = 12; and controls, *n* = 10) on day 10 postinfection. (A) Cytospin preparations were made and the BAL cells were differentially stained with Modified Wrights Stain. Numbers of neutrophils, macrophages, lymphocytes, and other cell types were determined by microscopy. Data are depicted as the means ± SEM of the relative frequencies of each population. There are significant differences in the relative frequency of alveolar macrophages (*P* < 0.003) and neutrophils (*P* < 0.002) in the airways of SCFP-treated animals compared with controls. (B) BAL cells were also analyzed by flow cytometry to determine the relative frequency of CD4 T cells (black bars), CD8 T cells (light gray bars), and γδ T cells (dark gray bars) in the airways. Data are presented as means ± SEM.

Neutrophilia is a hallmark of BRSV infection and the increased recruitment of neutrophils to the airways correlates with disease severity. As seen in Figure 6A, we observed differences in the relative frequency of alveolar macrophages (*P* < 0.003) and neutrophils (*P* < 0.002) in the airways of SCFP-treated animals compared with controls. The ratio of neutrophils to lymphocytes in the BAL also tended (*P =* 0.053) to be greater in control calves compared with SCFP-treated calves. The increased frequency of neutrophils in the lungs of control calves correlates with the observed increases in disease severity and lung pathology. Flow cytometry was used to identify relative frequencies of CD4, CD8, and γδ T cells present in the airways at this timepoint after infection. As seen in Figure 6B, SCFP-treated calves had increased (*P* = 0.038) frequencies of CD4 T cells present in the BAL fluid, and a trend (*P* = 0.085) toward increased frequencies of γδ T cells present in the BAL fluid. We also performed flow cytometry analysis of peripheral blood leukocytes at multiple timepoints both during the feeding period and after BRSV infection (Table 5). We observed a treatment × time effect for circulating neutrophils (*P* = 0.019), but no other effects of SCFP treatment on the relative frequencies of any circulating immune populations.

**Table 5.** Effects of SCFP treatment on the frequency of immune cells in blood in calves

Circulating immune cells, % of total live cells	Control mean	SCFP^1^ mean	SEM	Treatment effect *P-*value	Time effect *P-*value	Treatment × Time interaction *P-*value
CD4 T cells	14.31	14.50	1.50	0.223	0.0002	0.246
CD8 T cells	7.32	6.89	0.39	0.581	<0.0001	0.298
γδ T cells	17.61	15.39	1.86	0.310	0.0006	0.943
Neutrophils	17.30	18.83	1.02	0.527	<0.0001	0.019
Monocytes	7.06	8.08	0.40	0.221	0.0027	0.738
NK cells	2.17	2.11	0.09	0.556	0.0184	0.939

^1^Calves fed SCFP received 1 g/d SmartCare in milk and 5 g/d NutriTek top-dressed on the starter for 31 d.

### Innate immune responses

We observed no effect (*P* = 0.427 or *P* = 0.460, respectively) of SCFP treatment on phagocytic activity by peripheral blood neutrophils or monocytes at any timepoint (Table 6). Supplementation with SCFP also had no effects (*P* = 0.119 or *P* = 0.414, respectively) on the respiratory burst activity of blood neutrophils or monocytes at any timepoint (Table 6), although we did note a treatment × time effect for monocyte respiratory burst activity (*P* = 0.013).

**Table 6.** Respiratory burst and phagocytic activity by innate immune cells from blood (days 1, 10, 14, and day 3 postinfection) and BAL (day 14 of the feeding period and day 10 postinfection) of calves supplemented with or without SCFP products for 31 d

	DHR^1^ activity, ΔMFI^2^						Phagocytic activity, ΔMFI					
Blood	Control	SCFP^3^	SEM	Treatment effect *P-*value	Time effect *P-*value	Treatment × Time interaction *P-*value	Control	SCFP	SEM	Treatment effect *P-*value	Time effect *P-*value	Treatment × Time interaction *P-*value
PMN	45,036	56,239	9,415	0.119	<0.0001	0.785	306	285	142	0.427	0.196	0.437
Monocyte	42,072	31,038	8,586	0.414	<0.0001	0.013	355	430	76	0.460	0.010	0.280
BAL												
PMN	60,147	86,385	13,528	0.316	0.0004	0.121	2,294	4,532	1,070	0.152	0.296	0.891
Macrophage	37,053	68,962	13,048	0.113	<0.0001	0.055	6,232	9,797	1,634	0.0006	0.031	0.366

^1^DHR activity represents respiratory burst.

^2^MFI, mean fluorescence intensity.

^3^Calves fed SCFP received 1 g/d SCFP in milk and 5 g/d SCFP top-dressed on the starter for the 31 d.

Local immunity in the lung is critical for the prevention and control of respiratory infections such as BRSV. Therefore, we also determined the effects of SCFP treatment on innate immune cells recovered from the BAL. Samples were collected from the BAL once during the feeding period (day 14 of the study) and once on day 10 postinfection during necropsy. Interestingly, we observed an increase in phagocytic activity (*P =* 0.0006) by BAL macrophages from SCFP-treated calves compared with controls (Table 6). However, we observed no differences in their respiratory burst activity nor did we observe differences in BAL neutrophil phagocytic activity (*P =* 0.152) or respiratory burst activity (*P* = 0.316 for neutrophils and *P =* 0.113 for macrophages; Table 6).

**Figure 7.**
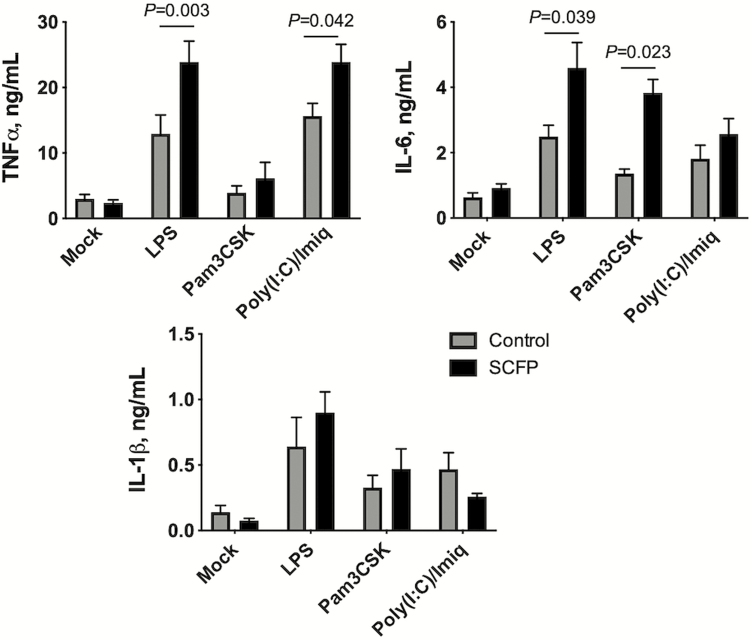
Enhanced inflammatory cytokine production by PBMCs from SCFP-treated calves compared with controls. PBMCs were isolated from peripheral blood on day 19 of the study (day 0 prior to BRSV infection) and cryopreserved. PBMCs were plated at 1 × 10^6^ cells/well in 24-well plates and stimulated for 72 h with 1 μg/mL LPS, 10 μg/mL Pam3CSK4, or a mixture of 50 μg/mL Poly(I:C) with 10 μg/mL imiquimod. Mock wells were treated with media only. After 72 h, cell culture supernatants were collected and analyzed by commercial ELISA kit for concentrations of IL-6, TNFα, or IL-1β; *n* =10 control calves; *n* = 12 SCFP calves.

Proinflammatory cytokine production is another critical aspect of innate immune function. Calves treated with SCFP mounted an enhanced cytokine response compared with cells from control calves. In response to LPS, the SCFP cells produced increased concentrations of TNFα (*P =* 0.003) and IL-6 (*P =* 0.039) compared with control cells (Figure 7). Cells from SCFP calves also produced more TNFα (*P =* 0.042) in response to Poly(I:C) and imiquimod, and more IL-6 (*P =* 0.023) in response to the TLR2 agonist, Pam3CSK (Figure 7 and Table 7). However, we observed no differences (*P =* 0.476 for LPS, *P =* 0.890 for Pam3CSK, *P =* 0.668 for Poly[I:C] and imiquimod) in the capacity of the SCFP cells to produce IL-1β compared with control cells at this timepoint. The responses to LPS stimulation across all timepoints during the feeding period and postinfection are depicted in Table 7. We observed treatment × time interactions and treatment effects throughout the feeding period, with PBMCs from SCFP-treated calves producing more TNFα (*P* = 0.096 and *P* = 0.0017, respectively) and IL-6 (*P =* 0.053 and *P* = 0.025, respectively) than PBMCs from control calves (Table 7). A treatment × time effect (*P =* 0.0024) was noted for IL-1β secretion on day 1 of the feeding period, but there were no overall treatment effects during the feeding period (*P =* 0.133). Treatment with SCFP had no effects on innate cytokine production following BRSV infection (TNFα, *P =* 0.532; IL-6, *P =* 0.087; IL-1β, *P* = 0.128). The responses to Pam3CSK4 and Poly(I:C)/imiquimod are depicted in Supplementary Tables S1 and S2, respectively. As with LPS treatment, we observed treatment × time interactions and treatment effects in the response to both stimuli (Supplementary Tables S1 and S2).

**Table 7.** LPS-induced inflammatory cytokine production by innate immune cells from blood (days 1, 7, 14, and 19 of the feeding period; days 3 and 7 postinfection) and BAL (day 14 of the feeding period and day 10 postinfection) of calves supplemented with or without SCFP for 31 d

	Blood						BAL			
	Control	SCFP^1^	SEM	Treatment effect *P-*value	Time effect *P-*value	Treatment × Time interaction *P-*value	Control	SCFP	SEM	Treatment effect *P-*value
Feeding period^2^										
TNFα, ng/mL	12.09	18.24	4.91	0.0017	<0.0001	0.096	9.86	4.23	1.74	0.028
IL-6, ng/mL	1.79	2.95	0.60	0.025	0.0002	0.053	3.78	1.98	0.61	0.039
IL-1β, ng/mL	1.04	1.36	0.50	0.133	<0.0001	0.0024	6.65	2.26	1.29	0.071
Postinfection^3^										
TNFα, ng/mL	20.29	17.41	3.16	0.532	0.341	0.675	23.18	15.26	2.81	0.033
IL-6, ng/mL	1.40	1.03	0.37	0.087	0.154	0.170	3.39	1.54	0.54	<0.0001
IL-1β, ng/mL	0.38	0.25	0.10	0.128	0.586	0.056	2.67	2.13	1.60	0.961

^1^Calves fed SCFP received 1 g/d SCFP in milk and 5 g/d SCFP top-dressed on the starter for the 31 d.

^2^Blood cells were collected and assayed on days 1, 7, 14, and 19 of the feeding period. Antemortem BAL samples were collected and assayed on day 14 of the feeding period.

^3^Blood cells were collected and assayed on days 3 and 7 after BRSV infection. Postmortem BAL samples were collected and assayed on day 10 after BRSV infection.

**Figure 8.**
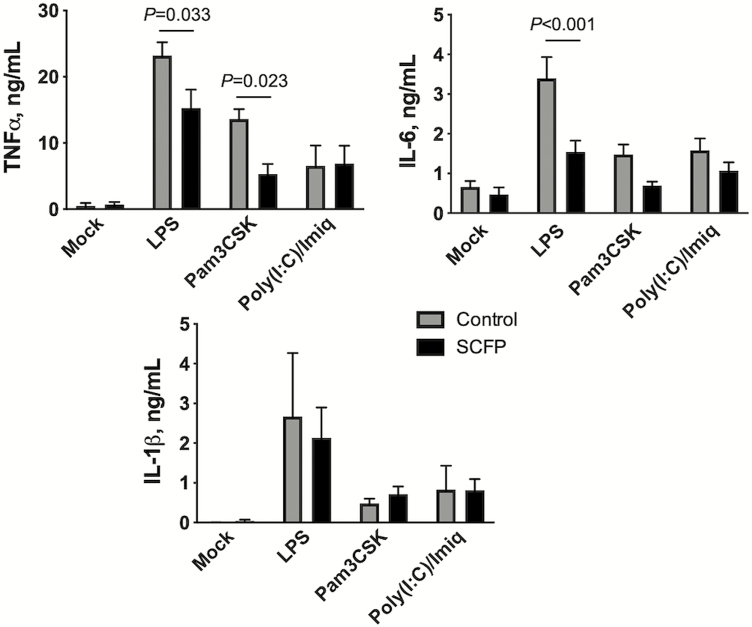
Reduced inflammatory cytokine production by BAL cells from SCFP-treated calves compared with controls. BAL samples were collected on day 10 after BRSV infection and cryopreserved. BAL cells were thawed, plated at 1 × 10^6^ cells/well in 24-well plates, and stimulated for 72 h with 1 μg/mL LPS, 10 μg/mL Pam3CSK4, or a mixture of 50 μg/mL Poly(I:C) with 10 μg/mL imiquimod. Mock wells were treated with media only. After 72 h, cell culture supernatants were collected and analyzed by commercial ELISA kit for concentrations of IL-6, TNFα, or IL-1β; *n* = 10 control calves; *n* = 12 SCFP calves.

When we examined the capacity of BAL cells to produce inflammatory cytokines, we noted an opposing effect of SCFP treatment. Cells isolated from the BAL of SCFP-treated calves on day 10 after BRSV infection showed a reduced capacity to produce TNFα (*P =* 0.033) and IL-6 (*P <* 0.001) following TLR agonist stimulation (Figure 8). Similar results were observed by BAL cells isolated on day 14 during the feeding period (Table 7 and Supplementary Tables S1 and S2).

### Adaptive immune responses

**Figure 9.**
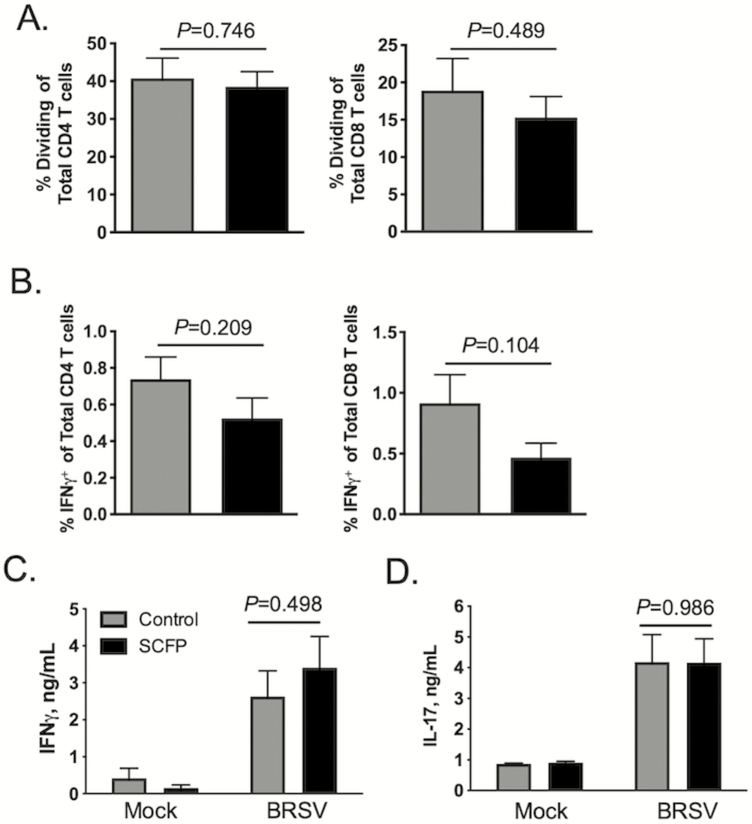
SCFP treatment does not alter BRSV-specific T cell responses in the blood. Peripheral blood was collected on day 10 after BRSV infection. PBMCs were isolated and cryopreserved. (A) PBMCs were thawed and labeled with CellTrace Violet proliferation dye, and 5 × 10^5^ cells/well were cultured for 6 d in the presence or absence of 0.01 MOI BRSV strain 375. CD4 (left) and CD8 (right) T cell proliferation was analyzed by flow cytometry for CellTrace dilution. Control wells remained unstimulated (Mock). T cell proliferation was analyzed using the gating strategy presented in Supplementary Figure S1. Background proliferation was subtracted, and results represent change over unstimulated samples; *n* = 10 control calves; *n* = 12 SCFP calves. Data are presented as means ± SEM. *P*-values were determined by student’s *t-*test. (B) Virus-specific IFNγ production was analyzed in PBMCs on day 10 postinfection; 1 × 10^6^ cells/well were stimulated in vitro with 0.01 MOI BRSV strain 375 for 16 h. Cells were then stained for intracellular IFNγ expression and analyzed by flow cytometry using the gating strategy outlined in Supplementary Figure S2. The frequency of circulating CD4^+^ IFNγ ^+^ (left) and CD8^+^ IFNγ ^+^ (right) is shown. Background IFNγ ^+^ production was subtracted and results represent change over unstimulated samples; *n* = 10 control calves; *n* = 12 SCFP calves. Data represent means ± SEM. *P*-values were determined by student’s *t-*test. (C and D) Cell culture supernatants were collected from the PBMCs cultures in (A) and analyzed by commercial ELISA kit for (C) IFNγ and (D) IL-17; *n* = 10 control calves; *n* = 12 SCFP calves. Data represent means ± SEM.

We next determined the effects of SCFP treatment on the adaptive immune response in the peripheral blood and lungs. As seen in Figure 9A, SCFP treatment had no effect on the frequency of antigen-specific CD4 (*P =* 0.746) or CD8 (*P =* 0.489) T cells that proliferated in response to BRSV stimulation. The capacity for IFNγ production was also evaluated using intracellular cytokine staining (Figure 9B). Representative gating strategies for intracellular cytokine staining are depicted in Supplementary Figure S2. We observed no differences in the frequencies of circulating virus-specific CD4^+^IFNγ ^+^ (*P =* 0.209) or CD8^+^IFNγ ^+^ (*P =* 0.104) cells between SCFP and control calves (Figure 9B). Consistent with the results of our intracellular cytokine staining, we observed no differences in virus-specific IFNγ (*P =* 0.489) production between SCFP and control calves (Figure 9C). We similarly detected no differences in virus-specific IL-17 (*P =* 0.986) production by PBMCs from SCFP-treated calves compared with control PBMCs (Figure 9D). Thus, SCFP treatment did not appear to impact the magnitude of the systemic, virus-specific cellular immune response.

**Figure 10.**
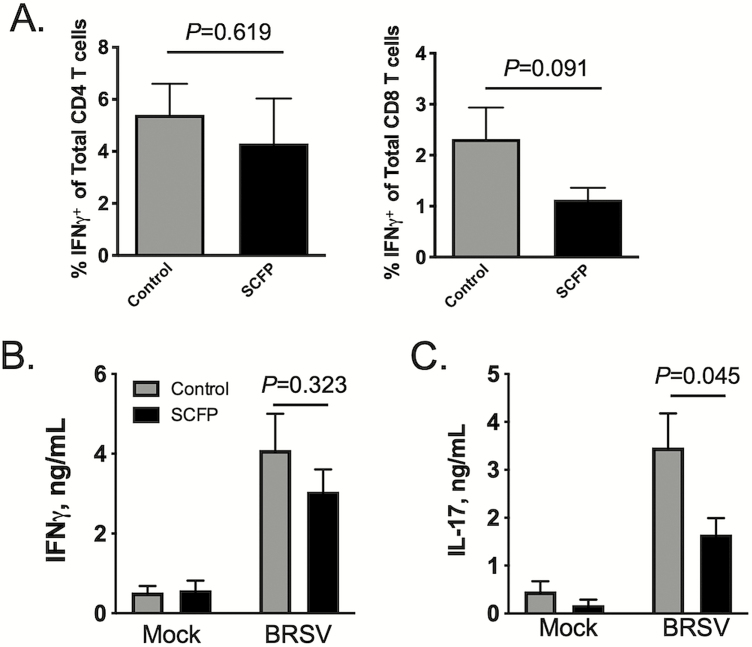
Impact of SCFP treatment on BRSV-specific T cell responses in the BAL. BAL samples were collected from all calves (SCFP treated and controls) on day 10 postinfection and cryopreserved. (A) BAL cells were thawed and 1 × 10^6^ cells/well were stimulated in vitro with 0.01 MOI BRSV strain 375 for 16 h. Cells were then stained for intracellular IFNγ expression and analyzed by flow cytometry using the gating strategy outlined in Supplementary Figure S3. The frequency of circulating CD4^+^ IFNγ ^+^ (left) and CD8^+^ IFNγ ^+^ (right) is shown. Background IFNγ ^+^ production was subtracted and results represent change over unstimulated samples; *n* = 10 control calves; *n* = 12 SCFP calves. Data represent means ± SEM. *P*-values were determined by student’s *t-*test. (B and C) BAL cells were thawed and plated at 1 × 10^6^ cells/well and stimulated in vitro with 0.01 MOI BRSV strain 375 for 72 h. Culture supernatants were collected and analyzed by commercial ELISA kit for (B) IFNγ and (C) IL-17; *n* = 10 control calves; *n* = 12 SCFP calves. Data represent means ± SEM.

Although we observed no treatment effects (*P =* 0.619) on the frequency of CD4^+^IFNγ ^+^ cells in the BAL (Figure 10A), there was a trend (*P =* 0.091) toward a reduction in the frequency of CD8^+^IFNγ ^+^ cells in the BAL of SCFP-treated calves compared with controls (Figure 10A). Supplementation with SCFP had no effect (*P =* 0.323) on virus-specific IFNγ production (Figure 10C) by BAL cells recovered on day 10 postinfection but did result in reduced (*P =* 0.045) production of virus-specific IL-17 (Figure 10C).

## Discussion

In the current study, PBMCs from SCFP-treated calves had an increased capacity for proinflammatory cytokine production in response to TLR stimulation; yet, we observed an opposing effect of SCFP supplementation on inflammatory cytokine secretion by mucosal immune populations. Consistent with this result, yeast fermentation products have been shown to have complex, and somewhat paradoxical, effects on immune function in other species. In dogs, SCFP supplementation results in increased circulating populations of major histocompatibility complex II positive antigen-presenting cells and activated IFNγ-secreting CD4 T cells, but an overall reduction in total white blood cells (Lin et al., 2019). Peripheral blood cells from SCFP-supplemented dogs produce reduced quantities of TNFα and IL-6 in response to TLR stimulation (Lin et al., 2019). In accordance with our own data, in an LPS challenge model, SCFP-supplemented piglets were shown to produce enhanced quantities of serum IL-6 and TNFα compared with controls (Burdick Sanchez et al., 2018). In humans, SCFP are inclined to have an overall anti-inflammatory effect, reducing the symptoms of allergic rhinitis (Moyad et al., 2009), cold/flu infection (Moyad et al., 2008, 2010), and histamine-induced skin inflammation (Jensen et al., 2015); however, acute SCFP treatment results in the increased activation of circulating immune cells and increased serum IFNγ concentrations (Jensen et al., 2011). Thus, the contrasting pro- and anti-inflammatory effects of SCFP treatment observed in our animal model concur with prior reports, suggesting that the immunomodulatory effects of SCFP are dependent upon context.

Innate training has emerged as a mechanism by which cells of the innate immune system exist in an enhanced or “trained” state, which enables them to respond to pathogenic insults with increased proinflammatory cytokine secretion. This heightened state of responsiveness is induced primarily in myeloid cells (monocytes and macrophages) (Quintin et al., 2012; Kleinnijenhuis et al., 2014; Saeed et al., 2014; Netea et al., 2016) and is independent of adaptive immunity. To date, several live vaccines and a few specific microbial components have been shown to “train” the innate immune system, including the yellow fever vaccine, the measles vaccine, and the live attenuated *Mycobacterium bovis* Bacille Calmette–Guerin (**BCG**) vaccine. Innate training is caused by epigenetic reprogramming and alterations in basal intracellular metabolic pathways, which result in long-term changes in gene expression and cell physiology (Kleinnijenhuis et al., 2012; Quintin et al., 2012; Saeed et al., 2014). To date, there have been few reports investigating innate training in agricultural species. However, we have recently reported that vaccination with BCG is able to train the innate immune system in the neonatal calf, resulting in enhanced capacity for IL-6 and TNFα secretion by circulating PBMCs and monocytes (Guerra-Maupome et al., 2019). The yeast cell wall component, β-glucan, has been well described for its capacity to “train” the innate immune system in rodents and humans (Quintin et al., 2012; Cheng et al., 2014; Saeed et al., 2014). Our results from the current study suggest that SCFP treatment may be training or enhancing the systemic innate immune system in the calf in a similar fashion as the BCG vaccine or β-glucan treatment. One important aspect that is unclear in the context of this study is the duration of SCFP’s effects on innate function. By definition, innate training results in long-term changes to innate immune function, with some reports in humans, suggesting that the innate immune system can exist in this heightened state for nearly a year (Kleinnijenhuis et al., 2012). In our hands, the effects of BCG vaccination on innate immune function in the calf endured for at least 3 mo (Guerra-Maupome et al., 2019). Here, the SCFP supplement was administered for the duration of the study; therefore, it is unknown if the effects of treatment on innate immune function would endure following the withdrawal of the supplement. Furthermore, due to the relatively short duration of this study, the long-term effects of SCFP supplementation on cattle health and performance remain unknown. Future studies are needed to shed light on the mechanisms by which SCFP treatment modulates innate immune function in the blood and the possible long-term nature of these effects.

It is increasingly recognized that a majority of the lung damage resulting from viral and bacterial infections during BRD is not caused by the pathogens themselves, but rather from an over-exuberant host immune response (Gershwin et al., 2005; Srikumaran et al., 2007; Watts and Sweeney, 2010). Neutrophil infiltration into the lung, effector T cells, and exacerbated production of inflammatory cytokines, the so-called “cytokine storm,” have all been implicated in increased pathology and poor disease outcome following BRD. Given its critical role in air exchange, the lung and airways are highly susceptible to immune-mediated damage. From studies in calves, it is clear that controlling the neutrophil influx into the lungs can positively affect the disease outcome. Depletion of neutrophils (Slocombe et al., 1985; Breider et al., 1988; Weiss et al., 1991), or inhibition of neutrophil infiltration to the respiratory tract (Caverly et al., 2001; Radi et al., 2001, 2002), prior to *M. haemolytica* infection can reduce gross pulmonary lesions, alveolar edema, and necrosis of alveolar septae. Similarly, during BRSV infection, neutrophil recruitment correlates with increased disease severity and poor outcome (Hägglund et al., 2017). IL-17 is a master regulator of neutrophil recruitment and activation. It activates stromal cells to produce chemokines, such as CXCL1, CCL20, IL-6, and IL-8, which subsequently induce neutrophil recruitment (Bystrom et al., 2013). IL-17 is predominantly produced by antigen-specific CD4 T cells, termed Th17 cells. We have previously observed that IL-17 is significantly upregulated in the lungs of calves infected with BRSV (McGill et al., 2016), and, in a recent report, noted that the use of a small molecule inhibitor to block IL-17 production resulted in reduced BRSV-associated lung pathology and improved disease outcome (McGill et al., 2019a). In our current study, BAL cells from SCFP-treated calves released reduced quantities of virus-specific IL-17 upon antigen restimulation. Consistent with reduced IL-17 production, we also observed reduced neutrophil infiltration in the BAL of SCFP-treated calves. Thus, SCFP may be affecting Th17 cell differentiation, resulting in positive downstream effects on the inflammatory milieu in the lungs. We noted increased frequencies of CD4 T cells in the airways of SCFP-treated calves compared with control calves. The cytokine profile of these helper T cells is unclear. Given that SCFP-treated BAL cells produced similar quantities of virus-specific IFNγ, but reduced quantities of IL-17, we speculate that the cells may be regulatory or Th2 cells. We performed ELISAs for IL-13 and IL-4 on cell culture supernatants from BAL and PBMCs cultures that were restimulated with BRSV, but quantities of these cytokines were below the limit of detection for the assay. Thus, it seems unlikely that SCFP-treated calves are mounting a strong Th2 response in the BAL. Future investigations should be aimed at characterizing the effects of SCFP treatment on T helper cell differentiation and cytokine production in the respiratory tract.

**Figure 11.**
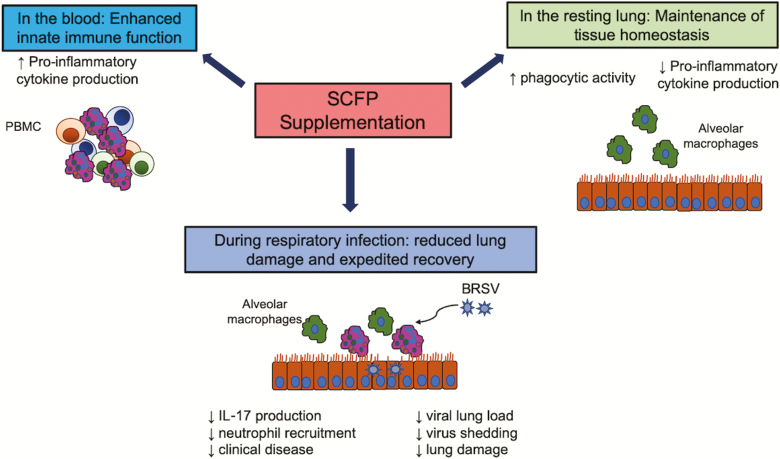
Our proposed model for the effects of SCFP treatment on immune function in the periphery and lung tissue. The results of our studies suggest that SCFP supplementation differentially modulates immune function in the periphery and lung tissue. In the blood, SCFP treatment results in enhanced innate immune function. Circulating PBMCs have an increased capacity to secrete proinflammatory cytokines, suggesting that they are poised to respond more robustly to an infection or insult. The essential function of the lung is air exchange. Exacerbated inflammation causes tissue damage and interferes with lung function; therefore, immune responses in the mucosa must be carefully controlled. In the current study, SCFP treatment resulted in reduced proinflammatory cytokine secretion following TLR stimulation but increased the capacity of alveolar macrophages to phagocytose particles, suggesting that they may have improved capacity to eliminate invading pathogens or particulates. SCFP treatment during BRSV infection resulted in less clinical disease, less neutrophil recruitment, less lung pathology, and reduced virus shedding.

In summary, treatment with SCFP resulted in reduced clinical disease, reduced lung pathology, and reduced incidence of secondary bacterial infection following an experimental BRSV infection. Calves on SCFP supplements also shed less BRSV, rendering them less likely to spread the disease, which may have positive implications for the health of the herd. Figure 11 depicts a summary of our results and our model by which SCFP treatment appears to be affecting calf health and immune function in the present study. The SCFP treatment enhanced innate cytokine production by PBMCs stimulated with TLR agonists but had an immunoregulatory effect on innate immune function by cells in the airways. Supplementation also reduced virus-specific IL-17 secretion by cells isolated from the airways, leading to reduced neutrophil recruitment to the lungs. In the context of respiratory disease, limiting inflammation in the lungs is critical for minimizing lung tissue damage and promoting rapid recovery. We speculate that SCFP’s effects of limiting IL-6, TNFα, and IL-17 secretion in the mucosa led to reduced neutrophil recruitment, reduced tissue necrosis, and gross lung pathology. Future studies should be aimed at further characterizing the immunomodulatory features of SCFP on respiratory health and immunity.

## Supplementary Material

skaa252_suppl_Supplementary_Material

## Abbreviations

BAL: bronchoalveolar lavage
BCG: Bacille Calmette–Guerin
BRD: bovine respiratory disease
BRSV: bovine respiratory syncytial virus
BW: body weight
DHR-123: dihydrorhodamine-123
DMSO: dimethyl sulfoxide
FBS: fetal bovine sera
IFNγ: interferon gamma
IL: Interleukin
LPS: lipopolysaccharide
MFI: mean fluorescence intensity
PBMC: peripheral blood mononuclear cells
PMN: polymorphonuclear leukocyte
SCFP: *Saccharomyces cerevisiae* fermentation products
TNFα: tumor necrosis factor-alpha
