# Supplementary material for: Supplementing a Saccharomyces cerevisiae fermentation product modulates innate immune function and ameliorates bovine respiratory syncytial virus infection in neonatal calves
Source: J Anim Sci. 2020 Aug 11;98(8):skaa252. doi: 10.1093/jas/skaa252 (PMC7457959; doi:10.1093/jas/skaa252)
Supplement: skaa252_suppl_Supplementary_Material [file skaa252_suppl_supplementary_material.docx]

**Supplementing a *Saccharomyces cerevisiae* fermentation product modulates innate immune function and ameliorates bovine respiratory syncytial virus infection in neonatal calves**

Asmaa H.A. Mahmoud^1,2^, Jamison R. Slate^1^, Suyeon Hong^1^, Ilkyu Yoon^3^, Jodi L. McGill^1^*

^1^Department of Veterinary Microbiology and Preventive Medicine, Iowa State University, Ames 50010

^2^ Animal Health Research Institute, Agricultural Research Center, Egypt

^3^Diamond V, Cedar Rapids, IA 52404

*Corresponding Author: [jlmcgill@iastate.edu](mailto:jlmcgill@iastate.edu)

Running Title: SCFP supplementation moderates BRSV disease

Key Words: *Saccharomyces cerevisiae* fermentation products, bovine respiratory disease, bovine respiratory syncytial virus, innate immunity

**Supplemental Figure 1**


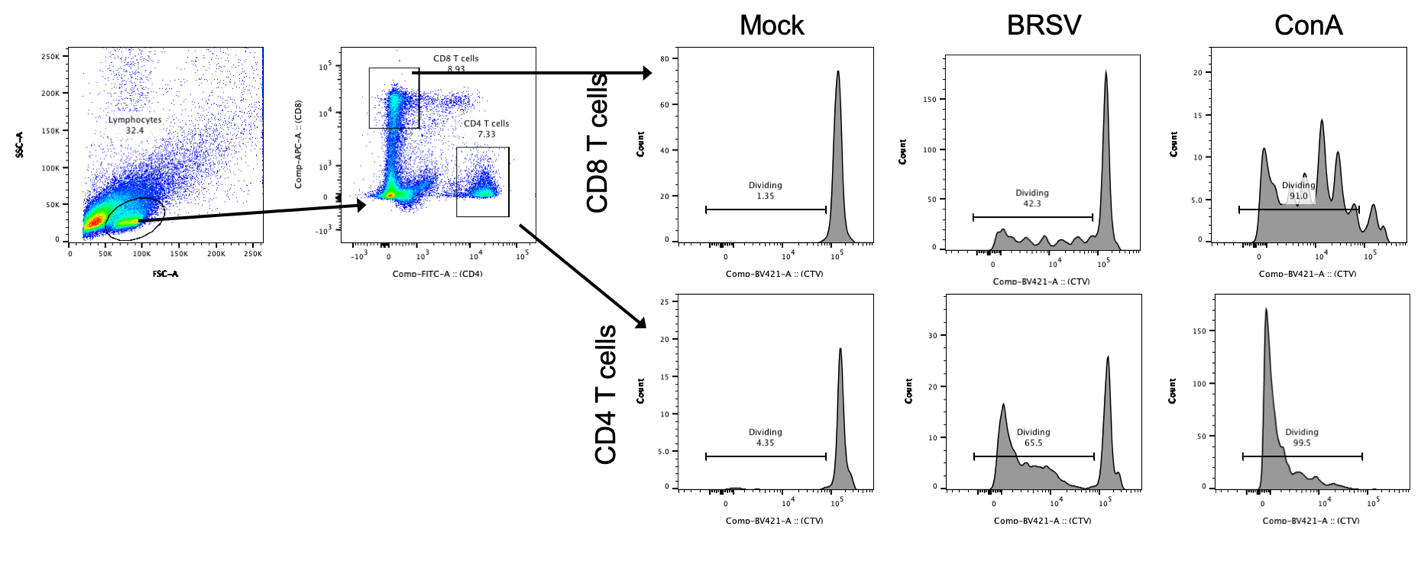


Supplemental Figure 1. Gating strategy for analysis of virus-specific CD4 and CD8 T cells in peripheral blood. PBMCs from control or SCFP treated calves were labeled with CellTrace Violet, and 5x10^5^ cells/well were cultured for 6 days in the presence or absence of 0.01 MOI BRSV strain 375. Positive control wells were stimulated with Concanavalin A. Negative control wells remained unstimulated. Cells were labeled with anti-bovine CD4 or CD8 and analyzed by flow cytometry for CellTrace dilution. Gating hierarchy (gating sequence as depicted by the arrows): single cells (now shown), lymphocytes (SSC-A vs FSC-A), CD4 or CD8 T cells and CellTrace dilution. Analysis was performed with Flowjo software.

**Supplemental Figure 2**


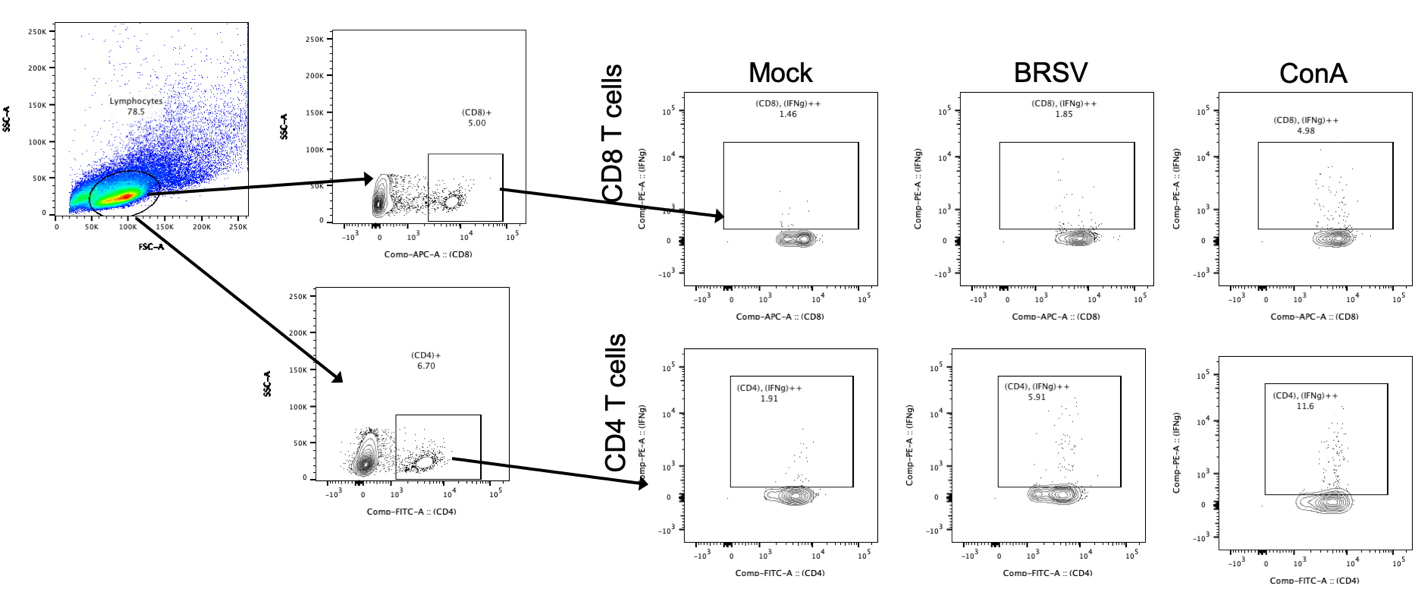


Supplemental Figure 2. Gating strategy for analysis of virus-specific, IFNγ-producing CD4 and CD8 T cells in peripheral blood. PBMCs from control or SCFP treated calves were plated at 1x10^6^ cells/well and stimulated for 16 hours with 0.01 MOI BRSV strain 375. Positive control wells were stimulated with Concanavalin A. Negative control wells remained unstimulated. Cells were labeled for 30 minutes with Live/Dead Aqua (ThermoFisher), then surface stained with anti-bovine CD4 or CD8, stained for intracellular IFNγ and analyzed by flow cytometry. Gating hierarchy (gating sequence as depicted by the arrows): singlets (not shown), lymphocytes (SSC-A vs FSC-A), CD4 or CD8 T cells and IFNγ expression. Analysis was performed with Flowjo software.

**Supplemental Figure 3**


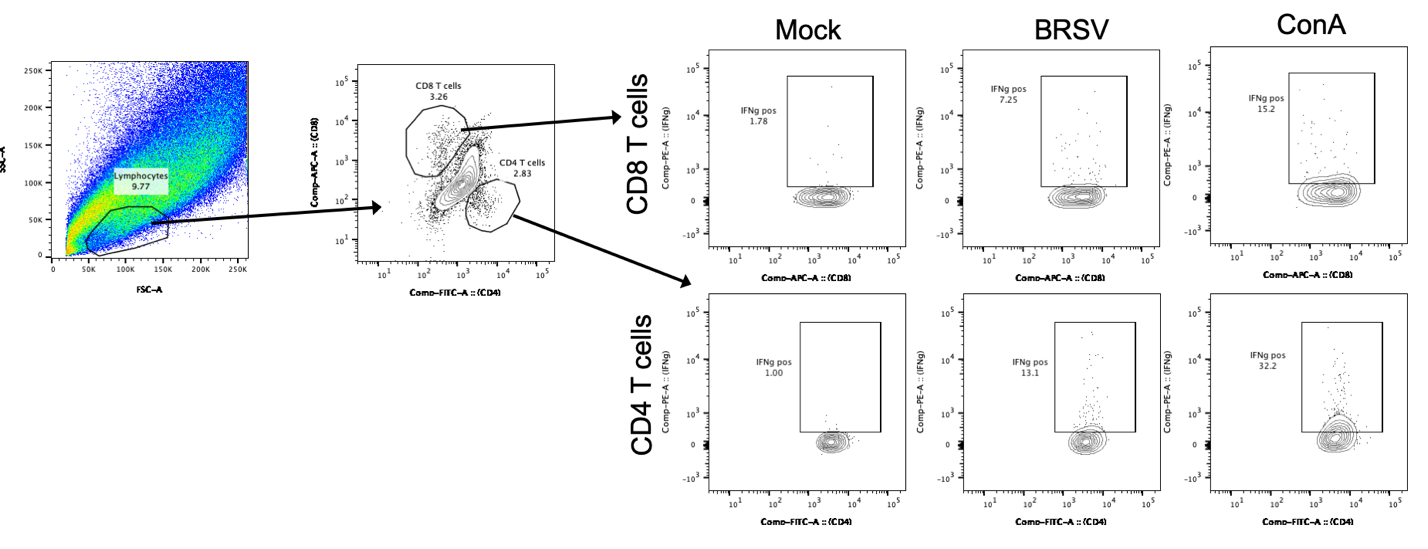


Supplemental Figure 3. Gating strategy for analysis of virus-specific, IFNγ-producing CD4 and CD8 T cells in the BAL. BAL cells from control or SCFP treated calves were plated at 1x10^6^ cells/well and stimulated for 16 hours with 0.01 MOI BRSV strain 375. Positive control wells were stimulated with Concanavalin A. Negative control wells remained unstimulated. Cells were labeled for 30 minutes with Live/Dead Aqua (ThermoFisher), and then surface stained with anti-bovine CD4 or CD8, stained for intracellular IFNγ and analyzed by flow cytometry. Gating hierarchy (gating sequence as depicted by the arrows): single cells (not shown), lymphocytes (SSC-A vs FSC-A), CD4 or CD8 T cells and IFNγ expression. Analysis was performed with Flowjo software.

Supplemental Table 1. Pam3CSK4-induced inflammatory cytokine production by innate immune cells from blood (d 1, 7, 21 of the feeding period; and d 3 and 7 post infection) and BAL (d 14 of the feeding period and d 10 post infection) of calves supplemented with or without SCFP for 31 d

|  | **Blood** | | | | | | **BAL^1^** | | | |
| --- | --- | --- | --- | --- | --- | --- | --- | --- | --- | --- |
|  | Control | SCFP^2^ | SEM | Treatment effect  *P-*value | Time effect  *P-*value | Treatment x Time Interaction  *P-*value | Control | SCFP | SEM | Treatment effect  *P-*value |
| **Feeding period^3^** | | | | | | | | | | |
| TNFα, ng/mL | 4.05 | 5.59 | 0.42 | 0.084 | 0.975 | 0.534 | 3.05 | 1.10 | 0.87 | 0.034 |
| IL-6, ng/mL | 1.78 | 2.15 | 0.62 | 0.002 | <0.0001 | 0.0007 | 3.16 | 2.19 | 0.41 | 0.232 |
| IL-1β, ng/mL | 0.43 | 0.59 | 0.12 | 0.434 | 0.003 | 0.143 | 4.28 | 1.15 | 1.94 | 0.088 |
| **Post Infection^4^** | | | | | | | | | | |
| TNFα, ng/mL | 7.63 | 6.99 | 4.33 | 0.703 | <0.0001 | 0.969 | 13.60 | 15.26 | 2.81 | 0.023 |
| IL-6, ng/mL | 0.93 | 1.14 | 0.79 | 0.039 | 0.0005 | 0.175 | 1.48 | 0.69 | 0.25 | 0.165 |
| IL-1β, ng/mL | 0.37 | 0.20 | 0.22 | 0.015 | 0.0012 | 0.008 | 0.48 | 0.71 | 0.19 | 0.998 |

^1^ BAL, bronchoalveolar lavage cells

^2^ Calves fed *S. cerevisiae* fermentation products (SCFP) received 1 g/d SCFP in milk and 5 g/d SCFP top-dressed on starter for the 31 d

^3^ Blood cells were collected and assayed on d 1, 7, 14, and 21 of the feeding period. Antemortem BAL samples were collected and assayed on d 14 of the feeding period.

^4^ Blood cells were collected and assayed on d 3 and 7 after BRSV infection. Postmortem BAL samples were collected and assayed on d 10 after BRSV.

|  | **Blood** | | | | | | **BAL^1^** | | | |
| --- | --- | --- | --- | --- | --- | --- | --- | --- | --- | --- |
|  | Control | SCFP^2^ | SEM | Treatment effect  *P-*value | Time effect  *P-*value | Treatment x Time Interaction  *P-*value | Control | SCFP | SEM | Treatment effect  *P-*value |
| **Feeding period^3^** | | | | | | | | | | |
| TNFα, ng/mL | 10.44 | 15.61 | 5.24 | 0.002 | <0.0001 | 0.338 | 8.35 | 3.24 | 1.74 | 0.037 |
| IL-6, ng/mL | 1.13 | 1.18 | 0.69 | 0.128 | <0.0001 | 0.303 | 1.38 | 1.25 | 0.47 | 0.998 |
| IL-1β, ng/mL | 1.68 | 1.85 | 0.94 | 0.834 | <0.0001 | 0.252 | 3.68 | 0.81 | 1.54 | 0.136 |
| **Post Infection^4^** | | | | | | | | | | |
| TNFα, ng/mL | 29.0 | 22.11 | 10.41 | 0.275 | <0.0001 | 0.049 | 6.57 | 6.89 | 3.04 | >0.999 |
| IL-6, ng/mL | 0.99 | 0.55 | 0.48 | 0.029 | <0.0001 | 0.045 | 1.58 | 1.07 | 0.3 | 0.564 |
| IL-1β, ng/mL | 0.31 | 0.25 | 0.18 | 0.396 | <0.0001 | 0.025 | 0.82 | 0.81 | 0.61 | >0.999 |

Supplemental Table 2. Poly(I:C)/Imiquimod-induced inflammatory cytokine production by innate immune cells from blood (d 1, 7, 21 of the feeding period; and d 3 and 7 post infection) and BAL (d 14 of the feeding period and d 10 post infection) of calves supplemented with or without SCFP for 31 d

^1^ BAL, bronchoalveolar lavage cells

^2^ Calves fed *S. cerevisiae* fermentation products (SCFP) received 1 g/d SCFP in milk and 5 g/d SCFP top-dressed on starter for the 31 d

^3^ Blood cells were collected and assayed on d 7, 14, and 21 of the feeding period. No data were available from blood samples collected on day 1 of the feeding period. Antemortem BAL samples were collected and assayed on d 14 of the feeding period.

^4^ Blood cells were collected and assayed on d 3 and 7 after BRSV infection. Postmortem BAL samples were collected and assayed on d 10 after BRSV.
